# Bridging the gap: Responding to resident burnout and restoring well-being

**DOI:** 10.1007/s40037-020-00567-3

**Published:** 2020-02-10

**Authors:** Ana Hategan, Tara Riddell

**Affiliations:** 1 Department of Psychiatry & Behavioural Neurosciences, Division of Geriatric Psychiatry, Michael G. DeGroote School of Medicine, Faculty of Health Sciences, McMaster University, Hamilton, Ontario Canada; 2 Department of Psychiatry & Behavioural Neurosciences, Michael G. DeGroote School of Medicine, Faculty of Health Sciences, McMaster University, Hamilton, Ontario Canada

**Keywords:** Resident physician burnout, Physician wellness, Physician well-being, Resilience, Curriculum development

## Abstract

There is an increasing awareness of high burnout found among physicians. Resident physicians particularly face heightened stress due to inherent pressures of training in addition to systemic challenges common to healthcare. It is crucial that medical training programs and organizations create a culture which promotes physician well-being. We conducted an evaluation of a quality assurance pilot program aimed at creating a safe space for increasing burnout awareness and well-being among resident physicians. The program was voluntary, offered to psychiatry residents enrolled at McMaster University, and comprised an online resilience curriculum, peer groups, and wellness newsletters. Data analysis took place between December 15, 2018 and July 15, 2019. The educational goals were evaluated by outcome measures obtained over time in aggregated response data through residents’ anonymous survey feedback. All aspects of the triad received positive feedback, with peer groups being perceived as most helpful. Of all residents, 31% (*n* = 22) engaged in all three aspects of the program; the majority were female (83%) and senior residents (63%). While 48% reported burnout upon enrollment, there was an average 50% stress reduction perceived post-attendance. This project has shown that peer groups can make a difference in the daily experience of psychiatry residents at our institution.

## Background

Residents and practicing physicians are at heightened risk for burnout, such that this is a growing endemic [1]. Physicians generally make healthy personal choices; however, regarding mental health, they fare poorly when compared with the general population. In a systematic review and meta-analysis involving more than 17,000 physicians worldwide, the prevalence of depression or depressive symptoms amongst resident physicians was 29%, which is significantly higher than the general population [2]. This highlights a serious issue, with a multitude of contributing factors that place resident physicians at grave risk. This risk includes the fact that physicians face death by suicide at double the rate of the general population (about 400 physicians die by suicide annually in the United States, or about a physician a day) [3]. In trying to account for these alarming numbers, suggesting that physician psychological wellness is declining, it is imperative to identify when issues with mental health may arise in our profession.

The practice of medicine itself and the rigorous training process involved seem to be closely associated with these issues, as a study of matriculating medical students has shown lower rates of depression and higher resilience compared with age-matched college graduates [4]. Although they start out well, as medical school progresses, burnout emerges and then peaks in residency where upwards of 60% of residents report being affected [1, 5]. Following medical training, the average physician continues to have significantly higher rates of burnout and decreased satisfaction with work-life integration and well-being than professionals in other fields [6].

When left unaddressed, burnout can be devastating to one’s professional development and functioning, particularly as this pertains to patient care as well as physicians’ own health and safety [7]. Interestingly, experts have concluded that burnout among healthcare professionals is primarily caused by systemic factors rather than problems with personal resilience [7]. Therefore, it is perhaps the organizational pressures and work stressors physicians face that may play a role in impacting their health and well-being [7]. As the long-term health and well-being of residents is likely to be affected during training, increasing numbers of institutions are now pursuing wellness as a core organizational strategy. In fact, wellness and self-care is now considered a core competency by medical education accreditation bodies across North America [8, 9]. Although stress in the medical profession is inevitable, burnout can be prevented or mitigated. If organizations are to positively impact physician mental health, teaching resilience skills may not be the only answer, but it may help.

Here we aim to evaluate a program that could lead this cultural shift by integrating digital technology and in-person activities. Integrating web-based and other digital resources into residents’ training allows them to learn at their own pace, and supports the growing popularity of such programs [10]. This may hold promise regarding future teaching modalities to cope with stress and promote well-being. Peer groups, including Balint and Doctoring to Heal programs, have already shown the potential to improve coping and sense of community [11–14]. The need to belong is a psychological desire for quality relationships [15]. Evidence has shown that social relationships can profoundly influence our well-being in the way that negative social connections have been found to be correlated with burnout [16]. Thus, positive social relationships with others can be crucial to well-being in the practice of medicine. It is known that one of the key areas that contribute to vulnerability to burnout is one’s sense of community, connectedness, and meaning [11]. In this view, physician engagement can be viewed as an opportunity to build/strengthen the sense of community, which could be an ‘antidote’ to burnout [17]. Finally, residents often cite concerns around confidentiality should they disclose or seek help for their struggles [18]. Therefore, for any supports to be adopted, they must be psychologically safe.

## Description of the innovation

This quality assurance pilot program used mixed methods for an exploratory sequential design. In this well-being building program, three infrastructural supports were used to promote adaptive skills: a) an electronic curriculum on resident resilience, b) wellness newsletters, and c) peer groups. Tab. 1 outlines the key components and objectives of this program. Qualitative peer groups revealed residents’ descriptions and perceptions about the infrastructural supports designed to facilitate wellness and informed design of a mixed method quantitative and qualitative survey of residents’ engagement in these learning behaviors. The subsequent survey’s purpose was to quantify residents’ priorities and values related to current infrastructural supports to promote wellness and well-being, and mitigate stress and burnout so to guide the department on further interventions.

**Table 1:** The main components and objectives of the well-being building triad pilot program

Components	Description	Objectives
Peer groups	– Offered quarterly in person, voluntary, structured/semi-structured time of 90 minutes – Resident-facilitated and attended to promote confidentiality and security – Integrates aspects of peer-support, debriefing, Balint and Doctoring to Heal groups, as well as experiential relaxation exercises – Promotes safe sharing of experiences and processing of emotions related to the ups and downs of residency	1. Build awareness and knowledge about the importance of enhancing wellness and mitigating burnout, while also employing principles of adult-learning, granting authority to learn at their own pace and for their own needs. 2. Combat stigma and stress to foster trainees’ comfort in sharing and destigmatizing their experiences with chronic stress and burnout, while empowering them to explore new ways to approach concerns and challenges. 3. Build a community of support and strength by demonstrating that physicians are not alone in their experiences and forming a unified voice calling for change. 4. Advocate for cultural change by encouraging dialogue between the medical infrastructure and medical professionals to maintain a bidirectional, healthy, and lifelong learning process aimed at caring for both the public and its healers. 5. Promote sustainability in medicine by supporting the modern physician to participate in a more effective and tolerable manner when addressing the needs of a rapidly changing healthcare landscape
Electronic resilience curriculum	– https://respite.machealth.ca – Publicly available, online, voluntary, and can be accessed by trainees during optional teaching times on their own schedules – Founded on two core learning dimensions: *Know Yourself *and *Integrate New Lifestyles*, which respectively teach about burnout, and offer strategies to enhance resilience or combat stress and struggles in medicine – Utilizes a case-based approach, skill-building exercises and quizzes to promote reflection and solidify learning	
Wellness newsletter	– Distributed quarterly via group listserve – Designed for residents, but both faculty and residents contribute and receive – Utilizes positive psychology, as well as personal stories and experiences to address stigma and isolation	

The McMaster University medical school is an urban, research-intensive institution in Ontario, Canada. In 2019, McMaster University placed second in the world in The Times Higher Education Impact ranking that recognized the impact universities are making in their own countries and on a global scale including good health and well-being of students [19]. In December 2018, the school’s psychiatry department (a 5-year training program) launched its new well-being program, which emphasizes inquiry and longitudinal foundational learning aimed at mitigating stress and burnout during residency training.

This program was voluntary and offered to all psychiatry residents enrolled at McMaster University in the 2018–2019 academic year. All residents received unlimited, open access to the online curriculum (a resource guide containing exercises and interventions designed to educate and enhance resident physician well-being) throughout the academic year. Between December 15, 2018 and July 15, 2019, they also received a total of three quarterly electronic newsletters distributed through the group listserve. Residents were invited through the group listserve to participate in the two in-person peer groups included in this evaluation, which utilized convenience sampling. They received two electronic invitations, first a month prior to and then a week prior to the event. The group meetings were resident-only, peer-run, confidential debriefing sessions. The McMaster psychiatry program provided four 90-minute unprotected time slots after the educational activities on residents’ weekly academic day, with a stipend for refreshments for a period of 12 months. Discussions included perceived difficult topics around experiences within and outside their life as a resident. Through guided and open discussions led by a senior peer, residents were introduced to the concept of peer-support dynamics that spontaneously emerged early in the program within peer groups. Tab. 2 further outlines the structure and group process, as well as the topics and experiences offered. Following the conclusion of each group, residents who attended received two electronic prompts at weekly intervals to complete a post-group survey to elicit feedback, first immediately after the group meeting and then a week following the event as a subsequent reminder.

**Table 2:** Structure and process of the peer groups

Pre-group	– All psychiatry residents invited via email to participate in upcoming peer group one month prior to the event. – An email reminder is sent out, again to all residents one week prior to the event.
In-group	*1) Resident arrival* – Residents obtain refreshments and settle in, socializing with their peers. *2) Welcome and check in (5 mins)* – Resident facilitators and participants introduce themselves and check in with how they are presently feeling. *3) Orientation (5 mins)* – Rationale and purpose for the group is reviewed, along with other more formal supports and resources available should these be needed. – Ground rules are reviewed: a. Maintain confidentiality b. Be non-judgmental c. Allow equal air time d. Become engaged e. Be respectful *4) Group discussion and debrief (50 mins)* – Residents engage in round-table open group discussion about a previously selected theme. – Residents developed strategies to address stress and burnout in medicine through sharing of their own experiences, supporting and validating one another, and learning from one another’s wisdom, skills and resources to help mitigate challenges and maintain one’s wellness. – Topics include: a. Difficult or adverse events b. Feedback and failure c. Work-life balance d. Seeking help *5) Experiential session (15–20 mins)* – Designed to promote grounding and relaxation post-discussion. – Experiences include: a. Body scans b. Mindfulness meditations c. Loving kindness meditations d. Progressive muscle relaxation *6) Closing and check out (5–10 mins)* – All residents comment on how they are now feeling after the group and what they will be taking away from the session. – Reminders provided regarding post-group survey to elicit feedback and the date for the next session.
Post-group	– Immediately following the group residents who attended the session are provided with evaluations. Paper evaluations were initially used, which were later switched to an electronic survey for ease and convenience. – Residents currently receive an email thanking them for their attendance and with the post-group evaluation survey immediately post the event, and then a week later as a reminder.

## Evaluation

The preliminary educational goals were evaluated through survey collections over 7 months obtained immediately post attendance at quarterly peer groups. Data were obtained through residents’ anonymous survey feedback in aggregated form. Data analysis occurred concurrently with data collection for early analysis to iteratively inform subsequent data collection. Two evaluators (a fourth-year psychiatry resident (TR) and a faculty member (AH)) analyzed peer group data using thematic key analysis. Evaluators drafted 15 survey items that each mapped phases of a framework which assessed the perceived stress level, level of engagement, adjusting, and learning of new skills. Respondents rated their level of agreement for each quantitative item on a 10-point Likert-type scale (1 = strongly disagree, 10 = strongly agree) and provided free-form answers to qualitative items. The two evaluators independently coded the transcripts and reviewed the coded data to synthesize information into themes. Preliminary data collection took place between December 15, 2018 and July 15, 2019 until sufficient information was obtained about the infrastructural supports to inform program design. Evaluators shared a reflexive approach [20] to the data by sharing with one another their perspectives based on their role in the school and experience and how this influenced their feedback to the data. Responses were anonymous before collection and analysis. We calculated descriptive statistics (frequency and averages) for all 15 survey items characterizing residents’ perceptions of the value of the resources and activities related to evaluated framework.

In the email sent to residents, we indicated the voluntary nature of their participation in this quality assurance activity. In addition, we prefixed our survey with informed consent information. Survey respondents who decided not to take part after attending the informed consent information were instructed to exit the survey. As a quality assurance project, the project was exempt from review by the authors’ institutional review board.

Tab. 3 summarizes the main findings of the pilot program. The mean attendance rate to peer groups was 31% (*n* = 22) of the total psychiatry resident cohort, representing a sample from across the 5‑year program. The survey respondent rate was 85%. Of all participants, 63% were senior residents (postgraduate training year 3–5) and 48% reported currently feeling burned out. The online resilience curriculum and wellness newsletters were appreciated, and the in-person peer groups were extremely well received. In the narrative comments, 100% of participants described peer groups as ‘very helpful’ and would attend again. Thus, the return attendance rate at the peer groups grew by 57%. Finally, whereas 83% perceived that external factors influenced their well-being, there was a perceived average stress reduction of 50% among attendees. Participants learned that peer support is a tool that can be utilized to monitor their progress and create learning goals to cope with stress.

**Table 3:** Preliminary results of the well-being building triad pilot program

Measures	Findings (average rates)
Resident participation cohort	31% of total psychiatry residents
Resident attendance return rate	23%; attendance amongst the peer group grew by 57%
Gender of participants	83% females
*Pre-attendance*	
Currently experiencing burnout	48%
Currently coping with burnout (somewhat well—not well)	75%
External factors influencing well-being	83%
Familiarity with supports/resources (somewhat—not at all)	66%
*Post-attendance*	
Would attend a future peer-support group	100%
Felt that the peer groups should be facilitated and attended by residents only	100%
Felt experiential sessions to be effective	100%
Strongly agree/agree that they learned new tools and practices to cope with stress and combat burnout	100%
Strongly agree/agree with feeling more comfortable talking about stress and burnout and/or asking for help when needed	80%
Strongly agree/agree with feeling more knowledgeable about resources or supports available and how to access these	80%
Strongly agree/agree with feeling respected and supported by peers	100%
Highly interested in further promoting resident wellness	83%
Average stress reduction in attendees	50% (5.5/10 to 2.75/10)
Cited accepted strategies to cope with burnout	– Informal supports/activities: 100% (discussing with family/peers/staff, physical activity, social activities, mindfulness and spiritual practices, and taking vacation) – Formal supports: 0% (resident wellness programs and other formal medical or psychological treatment)
Cited benefits of the program	Relaxing environment/exercises; sharing experiences with others; feeling less alone; understanding of self-compassion and practical tools to increase it; discussing tips to staying well and seeking support

Several limitations of this analysis should be noted including the single setting, relatively small sample, and preliminary self-reported outcome measures. Pre-post data collection did not include surveys of those who did not choose to participate. The questionnaire utilized was part of the preliminary pilot testing and was a non-validated, home grown tool that our department had developed. Although stress reduction was perceived among attendees, multiple factors may have been at play; therefore, it was impossible to fully ascertain whether participation in the program was the only determining factor for the stress reduction. Another limitation was psychiatry as a specific specialty. Many physicians affected by burnout do not seek help. In a 2019 report, though nearly two-thirds of physicians who reported burnout or depression stated they had not sought care for these concerns, psychiatrists were in fact the ones most likely to seek help [21]. It would be interesting to explore how residents in other specialties experience burnout, and how the impact of stigma and self-stigma can lead residents to avoid accessing services to manage chronic stress and burnout. The lower participation of junior compared with senior residents is understandable as in our program junior residents have higher call duties and off-service rotation requirements; however, as these residents are at increased risk of burnout [22] future efforts should focus on strategies to promote accessibility and optimize their engagement in such endeavors.

## Reflection

The reality is that practicing physicians are burning out, and physicians in training are not doing any better as reflected in our findings. Burnout is not just about exhaustion, detachment and inefficiency (the three core elements of burnout), it is about loneliness and isolation as well [1, 3]. Burnout and isolation are adding to unprecedented levels of depression and some of the highest rates of suicide among any profession across North America [1, 2, 5, 6, 8]. Solutions for burnout management have been equivocal. Yet, the need for relief has led to this local intervention that may show promise.

Our well-being curriculum incorporated infrastructure supports through innovative electronic tools and peer-facilitated, in-person debriefing and coaching groups, with structured time for goal setting. Preliminary feedback from residents suggests that residents identified value of learning strategies, including from their peers, to increase their well-being. In those who attended the program, there was perceived subjective reduction in stress, suggesting that residents may have efficiently assimilated new practices to cope with stress and burnout. Interestingly, in contrast to formal supports, using informal supports or activities was associated with increased feelings of coping well with burnout (Tab. 3). In the narrative comments, many residents reported feeling as if they ultimately had permission and space to feel and see themselves and their peers in the same way they have been taught to see their patients, as a “whole person.” Mutual respect, eye contact, active listening, and conversational equity, among other required key attributes for attendance, gauged signals that their ideas, values, and contributions matter. The authors aspire to integrate this triad as part of the future core curriculum on resident well-being at their institution with protected time for these sessions to occur during academic days. The authors hope that this program will provide a helpful forum to discuss important issues with peers and provide a safe and confidential resource to help cope with residency challenges, stress, and burnout.

In this profession, we have to find a balance. Healthcare settings and training programs may jointly need to begin implementing innovative approaches that could lead the way to community building through collaboration and peer-support networks, perhaps by creating programs such as a “resident physician well-being hub.” Designing such programs, however, must involve learners to best understand their needs and ensure confidentiality, including from the perspective of job availability and licensing, as psychological safety is paramount when expressing such vulnerability.

In conclusion, further trial of this triad may better examine its concrete benefits. Even though there are ongoing struggles that we do not have answers to, it was clear that the program’s electronic resilience toolkit, wellness newsletter, and peer groups were valuable. Although peer groups cannot counter the root causes of a burnout crisis alone, it can help build an engaged community of support and offer regular moments of connection over shared values with resident peers as a source of meaningful catharsis and reflection. As the focus on managing burnout shifts towards a shared responsibility between physicians and the healthcare system [7], it is the hope that such initiatives may lead to a cultural shift by fostering more open dialogue, as well as empowering and supporting residents as they take responsibility for their own well-being so that they ultimately can reap the rewards of a more fulfilling and sustainable professional life. This aligns with emerging and growing evidence to support new interventions aimed at building a community of support and the importance this plays in addressing/mitigating burnout.
